# Hospital and Institutional News

**Published:** 1912-11-02

**Authors:** 


### HOSPITAL AND INSTITUTIONAL NEWS.

### INSURED PERSONS AND VOLUNTARY HOSPITALS: THE QUESTION OF THE WEEK.

We direct the earnest attention of our readers to the report on page 121 on "State *versus* Voluntary Hospitals." There is no doubt the crisis which will have to be faced in the beginning of the year is one which demands the immediate attention of the managers of every voluntary hospital in the United Kingdom. The question of free medical service and free medical treatment to insured persons in voluntary hospitals is one which cannot remain undecided. From the insured's point of view it is, we hope, certain that British subjects will not show less independence of spirit than the German artisans have displayed, in which case they will refuse to accept medical treatment, for which they and their employers have paid, as charitable relief at a voluntary hospital. The voluntary hospitals cannot, without an infringement of their constitution and a departure from the objects for which they were founded, grant such free medical relief to insured persons, and when the National Insurance Act comes into practical operation after January 15, the honorary medical and resident staff must decline to attend insured persons in the voluntary hospitals as free patients. The latter point was clearly brought out at a meeting of the committee of management of the Royal Portsmouth Hospital, at which a letter signed by every member of the honorary medical and surgical staff was read. The effect of that letter, which is only a sample of those written under similar circumstances to other hospital committees, was to point out that, from the date in question until the terms and conditions of administering medical relief under the Insurance Act have been approved by the medical profession, it will be impossible for any member of the honorary and resident medical staff, except in cases of urgent necessity, to render professional service to an insured person through the medium of any voluntary medical charity. We need not repeat what is urged in the article to which we have already referred, but we may point out that the present impasse, if wisely dealt with, can result in perpetuating the voluntary system of hospitals in this country and placing their financial position once and for all upon a much sounder basis than it has ever yet been possible to secure. The independence of the voluntary hospitals must be maintained. The principle upon which they stand will always gain for them the generous support of the English-speaking race. That principle is that they exist for the benefit of persons who, though able to support themselves and their family when well, cannot provide for themselves or their dependants the cost entailed by illness or accident. This is essentially a matter for the British Hospitals Association to deal with immediately by setting in motion the necessary machinery to secure an interview with the Prime Minister and the Chancellor of the Exchequer, and to offer the Government co-operation in the present emergency by undertaking to place a certain number of beds at the disposal of the Insurance Commissioners for insured persons, upon the business basis that the whole cost entailed by the treatment of such cases, plus 10 per cent. on such cost to defray the cost of medical attendance, shall be paid to each hospital by the approved society to which the insured person seeking such hospital treatment may belong. On this plan the independence of the voluntary hospital is secured and its principle safeguarded. The position of the voluntary hospital would be strengthened by the service which it would thus render to the State, hospital abuse must be eliminated to a material extent, and the way would be open for the establishment of a system of co-operation which would bring into direct relations, and the closest intercourse for administrative purposes, the whole of the agencies for the relief of the sick in London and throughout the country. Full details of the plan here proposed will be found in the pamphlet entitled "The Future of the Hospitals," published by The Scientific Press, 28 and 29 Southampton Street, Strand. We hope that the voluntary hospital managers will agree to tell the Government quite frankly that they have no objection whatever to afford facilities for the inspection of the wards which they may set aside for the reception and treatment of insured persons. No well-managed hospital can do other than agree to such inspection, and the spontaneous offer to welcome it must give additional strength and independence to the voluntary hospitals of every kind throughout the United Kingdom.

### "THE HOSPITAL" REDUCES AN ASSESSMENT.

A case of considerable interest to hospital authorities was recently settled before the Doncaster Board of Guardians. The Royal Infirmary, Sheffield, had, in 1910, presented to them by a generous donor an old country mansion, with 3½ acres of ground, at Conisborough, twelve miles from the city of Sheffield. The Board structurally altered the old house, formerly known as "The Priory," into a convalescent home, containing twenty beds, with suitable quarters for the nursing and domestic staff. The overseers of the parish fixed the assessment at £100, and the convalescent home committee instructed the secretary of the institution to appeal on their behalf against such assessment. At the hearing it was pointed out that all hospital assessments should be merely nominal, that in dealing with a voluntary hospital or its convalescent home, which in this case is merely an annexe to the infirmary, rating authorities should confine the assessment to that portion of the premises which was beneficially occupied—that is, the quarters of the porter and the domestic staff—and that no hospital should be assessed beyond the ratio of £1 per bed. The case of the Bath Hospital and Rawson Convalescent Home at Harrogate, whereby the assessment was reduced from £727 to £50, reported in The Hospital on January 28,

1911, was cited to the overseers, with the result that the assessment has been reduced from £100 to £30 gross, that sum including £10 on the portion of the premises occupied by the porter and his wife. In this connection a hospital secretary writes "to express his appreciation of the assistance rendered by The Hospital to hospital managers and secretaries. The case of the Bath Hospital and Rawson Convalescent Home, not being reported in the local press, might easily have escaped attention." We are glad to think that the evidence The Hospital offered upon this assessment had a very important bearing on the overseers' decision at the hearing of the appeal.

### THE LATEST NAMES OF RED CROSS VOLUNTEERS.

On Tuesday last three medical and surgical units, which had been raised and equipped by the British Red Cross Society, left for service with the Turkish Army at the front. The director-in-chief is Major Doughty-Wylie, whose wife accompanies him in charge of the nursing arrangements; and the medical men in charge of the three units, each of which consists of fifteen men as dressers and orderlies, were:—First unit: Mr. Page, Mr. H. L. Mann, and Mr. L. Bourdillon, all of St. Thomas's Hospital. Second unit: Dr. Anderson, Dr. F. R. Thornton, and Dr. R. C. Ward. Third unit: Dr. Appleyard, of the Bethlem Royal Hospital, Dr. Steel, and Dr. Gardner. The cost of raising and equipping the units, which amounted to £3,600, has been paid by Sir Ernest Cassel. The units were seen off by a very similar group to that which assembled on the platform for a similar purpose last week, and Surgeon-General Sir Benjamin Franklin, a member of the Balkan Relief Committee of the Red Cross Society, travelled with the units as far as Paris.

### PELLAGRA IN BRITAIN.

Under this somewhat alarming title publicity has been given in our foremost daily newspaper to the views of Dr. Sambon concerning the method of infection by which this formidable skin disease is propagated; and it is stated on the same authority that pellagra exists in England, Scotland, and the Shetland Islands. The form in which this startling announcement has been made is perhaps open to criticism; a casual reader might readily get the impression that the disease, though undetected, may be widespread in Britain. So, of course, it may be, though the evidence in favour of this idea is extremely exiguous. But it does not do to forget that a few years ago pellagra was thought to be non-existent in the United States, whereas now it is known to be endemic in some of them. So it does not do to entertain any prejudices or to be too positive about the state of affairs in Britain. With this note of warning it may be well to review briefly the available evidence in support of the existence of pellagra in this country. Three cases were discovered during the sixties of the last century in Scotland. A doubtful case was described in Cheshire about six years ago. Two certain cases and nine doubtful ones have been discovered in Scotland in the last three years. Two cases have occurred in London during the same time. To found upon this slender record the supposition that pellagra is really endemic in Britain is much too sweeping an inference. It may conceivably happen that further research will reveal more cases, perhaps many more; but until that has happened it is foolish to prejudge the matter or to raise a panic on the strength of the suspicions entertained by the author of a theory which is not yet generally accepted as proved.

### THE LISTER MEMORIAL.

The decision as to the form of the memorial to Lord Lister was announced at a representative meeting which was held at the Mansion House last week. The meeting approved the suggestion of the general committee for a threefold scheme—namely, a medallion in Westminster Abbey, a monument in a public place in London, and an International Lister Memorial Fund for the Advancement of Surgery. The first two sections of the scheme are to be taken as a matter of course, the third is the only one which is likely to excite controversy. The task of allocating public money of this character is one of great difficulty, because those entrusted with it naturally desire first to gain popular approval, while at the same time the things in which the public are interested are the very ones in least need of additional help. We note, however, as regards the International Lister Memorial Fund for the Advancement of Surgery, that "grants in aid of researches bearing on surgery or awards in recognition of distinguished contributions" will be made "irrespective of nationality." We can hope only that this clause will allow of sufficiently wide interpretation for the assistance of investigators and researchers in genuine need. It must be added, however, that pioneers are not generally recognised as such till their work is common property. Still, if the Fund is there, it may encourage aspirants, though Lister's work has made not merely modern surgery, but modern nursing, and even hospital construction its heirs, and in a true sense co-claimants.

### GUY'S HOSPITAL: A DEFENCE.

In our last week's issue we offered a short comment on the recent exacerbation of the chronic dispute between the authorities at Guy's Hospital and the Southwark Guardians. At that time only the case against the hospital had been heard; and we felt some hesitation, therefore, in forming or expressing the opinion that the casualty officers of the hospital had been remiss and unwise over their disposal of certain patients. Now, however, the superintendent of the hospital has sent to us a long reply on behalf of the Governors to the Southwark Guardians. This reply shows convincingly that, as we indicated last week, the root of the whole business is the failure of the Guardians to make proper provision for the sick poor of Southwark, and their attempt to shift their responsibilities on to Guy's Hospital. But it is absolutely silent concerning the individual cases whose merits have lately been agitating the daily press. Silence does not always mean consent; but it is often so interpreted by the many, and if the Governors of Guy's have anything material to say in defence of the specific cases of which complaint was made to them, it is a great pity that they should leave it unsaid. As matters stand, the impression that there were mistakes made in the casualty room is likely to gain ground and to obscure the real issues at stake.

### MEDICAL INSPECTION TAKEN SERIOUSLY.

THERE are two sides to the question of the medical treatment of school children which have been brought to light by the medical inspection of the past year or two. The first side, which we discussed recently in these columns, is that the children under school age require even more rigid care than their elders, though little enough is this recognised at present, and the second that medical inspection which is not succeeded by medical treatment is all but useless. This second point appears to have been thoroughly grasped by the Surrey Education Committee, which has just received a special report from the medical inspection sub-committee to the effect that "voluntary efforts had failed to such an extent that 58 per cent. of the recommendations to obtain treatment, made after medical inspection of the children, had not been followed." The committee, therefore, urged the establishment of school clinics, and recommended, in the first instance, that twelve clinics should be provided; that the county be divided into six areas, each with two clinics under the charge of a whole-time medical officer, each area to have about 11,000 children. It was suggested the salary of each of the six medical officers should be £400, with £50 for travelling expenses. Dental surgeons should be employed to give their whole time to the work, each at a salary of £300, with £50 for travelling expenses. The defects to be treated at the school clinics should be confined to ringworm and other skin diseases, defects of eyes and vision, defects of ears and hearing, defects of the nose and throat, and decayed teeth. Care committees should be established for each school or group of schools. The parents should be required to pay for the treatment. The committee urged that the Board of Education should be pressed for more definite information as to the amount of the grant to be made. The estimated maximum cost of the scheme was £7,050 a year, and the complete scheme £11,750 a year, from which should be deducted £1,800 at present spent on medical inspection only. The report was adopted and sent forward for the approval of the County Council.

### MORE LIGHT ON QUACKERY.

FURTHER light was thrown on the wiles of quack medicine-mongers by the assistant editor of *Truth* when he appeared before the Select Committee on Proprietary Medicines to continue his evidence. It would appear that the promise made by a certain class of charlatan to return the money to dissatisfied purchasers is rarely fulfilled, so that the business morality of the quack is even less pronounced than his scientific qualifications. Mr. Paternoster said that in regard to nearly all the so-called "cures," the guarantee which had been given in recent years —that the money would be returned if there was no cure—was not complied with. The usual plan adopted by the quack was to write saying that the sufferer had not followed his conditions, and very often a new supply of the "cure" was sent, with the suggestion that the sufferer should go on trying it. Speaking of the fortunes made in the secret-remedy business, the witness mentioned the case of a man who exploited a nostrum—consisting of sugar of milk—for the cure of drunkenness; this man spent something like £20,000 a year on advertisements, and when he died left a fortune of £80,000. Another case he mentioned was that of a man who, after plying his trade under many aliases, was arrested in Berlin on a charge of fraud, forfeited his bail of £5,000, and is now connected with a "cure" for deafness which is advertised in the English press. The evidence went to show, as the Chairman suggested in one of his questions, that there is an almost inexhaustible field of folly in which any clever advertiser can almost with certainty reap a rich harvest. To protect the public from its own folly Mr. Paternoster suggested that there should be a full disclosure of the active ingredients of the medicines, and that it should be made an offence at law to make any misstatements with regard to the history, contents, or effects of any remedy in the advertisement, on the label, or in printed circulars sent to any person with the object of inducing him to purchase it.

### DOCTORS AND THE VICTORIAN ORDER.

THE KING has been graciously pleased to make the following appointment to the Royal Victorian Order to take effect from the date noted:—"To be Member of the Fourth Class. 10th October, 1912. Henry Edgar William Hoffmeister, Esq., M.R.C.S." Mr. Hoffmeister, who is Surgeon in Ordinary to Princess Henry of Battenberg, is a prominent practitioner at Cowes. He graduated in arts and medicine at Cambridge, and afterwards studied at St. Bartholomew's Hospital. He is consulting physician to the Royal Convalescent Home for Officers at Osborne and surgeon to the Royal Yacht Squadron.

### A NEW MENTAL HOSPITAL SUPERINTENDENT.

DR. THOMAS PHILIP COWEN, senior medical assistant at the Prestwich Mental Hospital, who has been in the service of the Lancashire Asylums Board for twenty-one years, has been appointed by the Board to be medical superintendent at Rainhill Mental Hospital. Dr. Cowen, who had a singularly brilliant academic career, is an M.D. of London University, and first was a student and then held many posts at St. Thomas's Hospital.

### DEATH ATTRIBUTED TO A PAIN CREAM.

WE publish without comment the verdict and remarks of the jury at an inquest held at Weston on Monday last, before the Spalding Deputy Coroner, on the body of Jessie Salome Abbott, aged nine, the daughter of a farm foreman. According to the evidence the child when ill was given by her mother several doses of Nicholl's Pain Cream, and afterwards became worse and died two days later. The mother stated that she only gave the prescribed dose of five drops on four separate occasions, but it was alleged that she had made a statement to a witness that the dose given was half a teaspoonful. Dr. Bone gave evidence to the effect that death was due to an irritant poison. He stated that the doses of five drops would not have been sufficient to cause the child's death, but that the larger doses of half a teaspoonful might have produced fatal results. The jury returned a verdict that death was occasioned or accelerated by use of the medicine mentioned, and said that preparations of this character should not be obtainable from ordinary shopkeepers.

### THE CASE FOR THE MENTAL CLINIC.

In discussing the best preventive methods to adopt in dealing with incipient mental disease, Dr. Edwin Stephen Pasmore, medical superintendent of Croydon Mental Hospital, makes an eloquent plea for the establishment of mental clinics. After showing from tables that the majority of patients had been mentally afflicted for twelve months before their admission, Dr. Pasmore suggests that the lunacy authorities in every town should co-operate with the local hospital authorities. He proposes that a mental clinic, presided over by the medical officers of the local mental hospital, should be instituted at every local hospital in the country, where there is a mental hospital at hand, but so as not to clash with other out-patient departments. The cost, he thinks, could be borne by an arrangement between the lunacy authorities and the local hospital committee. If the medical officers from the mental hospital could attend on one or two mornings a week, Dr. Pasmore believes that people might be attracted to bring their friends on the first sign of any mental aberration to seek the special advice provided. This, he argues, would do away with the present difficulty of getting hold of incipient cases, the relatives and friends of whom are well known to dread seeking a mental specialist's advice from the horror they have lest a certificate of insanity and institutional treatment should follow. What certain London hospitals have tentatively undertaken might well be established on a larger scale throughout the country. Once, again, many towns on the Continent set England an example in this matter.

### SEX RIVALRY IN HOSPITAL ADMINISTRATION.

It is much to be regretted that the appeal for funds for the proposed South London Hospital for Women should have excited the old sex rivalry in medicine, which we all hoped had received its death-blow long ago. But with what object save to embitter controversy can Dr. Ernest Fothergill have allowed the following sentence to creep into a letter in last week's *Times*? "The chief reason (he says) for this proposed hospital seems to be . . . the provision of additional wards and beds for women physicians and surgeons apart from men. . . . This attempt to further accentuate in London hospital administration the differentiation of sex is retrograde and hardly a movement one would expect to be initiated by women at the present time." The heading of this paragraph must have struck every reader as an echo from the past, and that Dr. Fothergill should resurrect it now is worth recording as an oddity. For the sex question in hospital administration ceased to exist as soon as women had gained their long-sought admission to the ranks of medical practitioners, and as soon as their rights in this matter were recognised by the establishment of women's medical schools. When these changes had been made the question of sex in medicine ceased to exist in the same way as a legal question ceases to exist when decided by a tribunal from which there is no appeal. Dr. Fothergill is trying to draw a red-herring across the question of the new hospital in a manner that we trust will not escape the public. For it is an attempt to reopen a question which has been settled for the past ten years.

### THE NEW CHIEF SURGEON TO THE POLICE.

The vacancy created by the recent death of Mr. Clinton Dent attracted a large number of applicants for the post of Chief Surgeon to the Metropolitan Police Force. Nor was the competition severe only in point of numbers: there were several names well known in the profession amongst the candidates. The successful competitor is Mr. C. A. Ballance, M.V.O., M.S., F.R.C.S., surgeon to St. Thomas's Hospital, and consulting surgeon to the National Hospital for the Paralysed and Epileptic in Queen Square. Mr. Ballance's long connection with St. Thomas's and his extensive researches upon the healing of nerves and upon cerebral surgery have made his name familiar for many years. His appointment is an admirable one, for he combines long experience and an all-round surgical ability with a knowledge of affairs and men. He has been president of the Medical Society of London, is a member of the Council of the Royal College of Surgeons, holds a Prussian decoration, and is an honorary member of American and French surgical societies. That the Police can command the services of such eminent men as Mr. Dent and Mr. Ballance is most satisfactory.

### LESSONS FROM A GUARDIANS' MEETING.

The Gloucester Board of Guardians have provisionally accepted tenders for the building of the new infirmary, which has been discussed at their meetings for a considerable time. The new infirmary is designed to provide accommodation for 149 beds, and the architect is Mr. Walter B. Wood. The tender of Messrs. Byard and Company, a local firm, was the lowest sent in, and amounted to £25,990, but this does not include the architect's fees nor those of the clerk of the works, nor the cost of furnishing. These items are expected to bring up the total cost of the new infirmary to about £30,000. As an instance of the want of business sense which accounts for the dilatoriness from which schemes undertaken by Boards of Guardians sometimes suffer, we may mention that this last meeting, at which the tenders were discussed, was made use of by one member to move that the building of the infirmary be no longer proceeded with. The question, perhaps, has not received as much attention and care as its importance demands, but it is astonishing that the Guardian in question had not raised the arguments against it before this, if he were sincere in advancing them. Naturally and promptly the Chairman, Mr. W. Priday, ruled him out of order; but the want of business sense shown by the gentleman in question is one that does not always receive the castigation that the Chairman in this case gave to it. Reference was also made to the frequency of resignations, thirteen having been received from various officers in little more than six months. This usually indicates that particular defect in administration of which the above incident was an example. For there is no better test of good management in institutional work, from hospital administration to hotelkeeping, than the absence of change in the personnel of the staff.

### THE PROMOTION OF SCOTTISH MEDICAL OFFICERS OF HEALTH.

THE executive of the Scottish Medical Insurance Council, as the result of a meeting in Glasgow, has resolved to present certain strong criticisms to the Local Government Board for Scotland. Their objections concern the proposal made to appoint the medical officer of health as chief tuberculosis officer on the ground that it is opposed to the interest of tuberculosis patients. They argue that the chief tuberculosis officers should be men of wide experience in the clinical manifestations of tuberculosis, the sort of men, in fact, who would be trusted as consultants by the profession. There is, no doubt, a great deal to be said against the automatic translation of the medical officer of health into the chief tuberculosis officer, but it does not necessarily follow that the proposal should be condemned wholesale. Cases exist in which the medical officer of health is a man of wide clinical experience, and we have little doubt that cases could be found in which men "who would be trusted as consultants by the profession" would not be prepared to accept the post of chief tuberculosis officer. Since many Corporations have a sneaking knowledge that their medical officers are far from being overpaid, the opportunity here presenting itself of promoting them to these new positions may often be acted upon. That it should invariably be so would be a matter for criticism, but the proposals of the Scottish doctors are not so easy to act on as they are plausible to state.

### A NEW PROTECTIVE FOR X-RAY WORKERS.

THE practical results of a reported invention by which silk can be made impenetrable by the $x$-rays will be awaited with interest by radiologists. The need for a supple and thin protection for the hands of those exposed to the $x$-rays has long been felt. Though, perhaps, at the present time other means are in use for shutting off the rays except in the required direction, there is still a great demand for gloves and other coverings. Up to now gloves and aprons have been of far too solid consistency to be comfortable, and if this process, by which silk can be made to take up certain metallic compounds, proves to be a success in cutting off the $x$-rays the radiologists will not be slow in availing themselves of it.

### A DEFECT IN THE SCHOOL OF TROPICAL MEDICINE.

IN speaking at the annual dinner of the London School of Tropical Medicine last week Sir Ronald Ross drew attention to a serious defect in one department. He urged that the science of tropical sanitation should be greatly developed, and said that a course was needed in this branch which could be given to men who had already obtained the diploma or certificate of tropical medicine. Sir Patrick Manson said, in reply, that such a proposal had been contemplated more than once, and deprecated the suggestion that such a course of instruction might be carried out elsewhere than in the London School. We agree with him in thinking that anything which would weaken the School's position would be a public calamity, and that London is the proper centre for organising such a study, particularly now that last year's schemes are to be realised and that we may "expect to find in a very short time a school, not so worthy perhaps as it might be, but one very much better in regard to accommodation than the present institution."

### DOCTORS AND MUNICIPAL POLITICS.

OUR recent reports of two medical candidates for Parliament, both of whom have been put forward in the Labour interest, may be supplemented by news of a medical man having engaged in municipal politics at Bradford. In the forthcoming municipal election in that city Dr. W. S. Hall will contest the North Ward, and it may be noted that he again is the Socialist and Labour representative. In an interesting speech at the meeting which adopted him as candidate Dr. Hall emphasised the connection between municipal trading and the prevention of disease, affirming that the public control of the water supply had been accompanied by a great reduction of epidemic disease. He stated that in his opinion the high death-rate of the North Ward was due to contaminated milk, the supply being drawn from a thousand sources. Organisation and system would supply the way out, and should be obtained by applying municipalisation to milk as it had been applied already to water. Dr. Hall, who has had four years' experience as Poor-Law medical officer in Bradford, then proceeded to criticise the existing infirmary and to argue that, though the scheme for a new institution was a fine one, he thought it was always going to remain a scheme. Needless to add, the inference Dr. Hall drew from this was that the Council should take the matter in hand and municipalise the hospital. Perhaps he will not be so keen on this remedy when he has seen what State hospitals mean to the patients, as our articles on State hospitals in Germany are proceeding to show.

### A COMMITTEE'S "UNFAIR" QUESTIONS.

AT the monthly meeting of the executive committee of Worcester General Infirmary the election of a house physician caused some unusual discussion. The first candidate who appeared was asked by a member of the honorary staff if he had signed the pledge of the British Medical Association. The question was denounced as unfair by a lay member of the committee, and some heated discussion ensued. Finally, on the Chairman's decision, it was withdrawn. It is interesting to note that the question came from one of the medical staff and its denunciation from a lay committeeman.

### AMALGAMATION COMPLETED AT BOURNEMOUTH.

It will surprise many to learn that the amalgamation of the Royal Victoria and West Hants Hospitals was not complete till last week, but any hasty feeling of criticism will be removed when it is added that it is the lawyers who discovered the difficulty. Mr. D. H. W. Robson Burrows presided at a special meeting of governors, which approved a scheme submitted by them, on counsel's advice, to the Charity Commissioners, by whom no objection had been raised. The Charity Commissioners, explained Mr. Robson Burrows, had put into shape an Act of Parliament embodying the governors' suggestions. A new trust is being formed which will relieve the old trustees of their responsibility. The scheme has another interest in that it necessitates such alterations in the rules as would provide that no person be admissible to the out-patient department whose income exceeds £1 a week, nor any member of a family, the collective income of which at home exceeds 30s. 6d., though the Chairman used his influence to retard the immediate approval of the alterations on the ground that the relations of the medical profession to the Insurance Act was too undefined to warrant any action in the matter. Dr. Roberts Thomson said that the governors were to be congratulated that the scheme had turned out so simply for the hospital, and that the amalgamation had created a precedent, referring to the valuable work done by Mr. D'Angibau and Mr. Frances, and to the fact that only two other hospitals had done anything of a similar kind.

### AN ACTIVE NORTHERN SUPERINTENDENT.

We briefly noticed last week the appointment of Mr. E. L. Blake, secretary-superintendent of Oldham Infirmary, as president of the Manchester and Districts Branch of the Incorporated Association of Hospital Officers. The appointment is a happy one, if only because on the formation of this society Mr. Blake was one of its first members. He has had, in fact, a long and varied experience of hospital work. For twenty-five years he has held the position of honorary secretary of the Hospital Saturday Committee for the Oldham Royal Infirmary, and during this period has helped to raise over £50,000 from this source for this institution. During the last fifteen years he has held, and still retains, the position of superintendent-secretary. On his appointment the annual income of the infirmary from all sources was between £3,000 and £4,000; the income is now over £9,000 per annum, and the number of beds has increased from 70 to 130. There has also been erected an additional wing containing 60 beds, a new nurses' home recently opened at a cost of nearly £8,000, and a separate laundry. Mr. Blake also takes an active interest in the Friendly Society movement.

### THE RED CROSS AND THE WAR.

Two units of the British Red Cross Society have left for service in Greece under the command of Colonel Charles Delmé Radcliffe. The members assembled at the Millbank quarters of the Royal Army Medical Corps, where Sir Frederick Treves read to them a congratulatory and sympathetic message from Queen Alexandra. As showing the interest taken by the voluntary hospitals in this movement, we may note that Mr. H. V. Welch, the senior house surgeon at Derbyshire Royal Infirmary, has relinquished his appointment to proceed to the field of operations in the Balkans at the invitation of the Red Cross Society. The board of management gave him permission to accept the invitation, and he sailed for Greece last Sunday evening. The staff at the infirmary gave him an enthusiastic send-off.

### PHARMACISTS AND THE INSURANCE ACT.

From the point of view of pharmacists the Chancellor of the Exchequer's statement respecting the sum allotted for the payment of drugs for insured persons is not wholly unsatisfactory. It cannot be said, however, until we have some experience of the working of the scheme, whether the one and sixpence or even the two shillings per head will be sufficient to meet the cost; but pharmacists seem disposed to do the best they can with the arrangement. There can be no doubt that the scale of charges which the Pharmaceutical Standing Committee, in consultation with the Insurance Commissioners, is drawing up as a model for Insurance Committees will allow of only a very modest profit being made if the sum allowed is not to be exceeded. That part, at any rate, of the extra sixpence available for drugs will have to go to the chemist seems certain, when reference is made to the cost of this part of the scheme in Germany, even when due allowance is made for the "extras" which the German societies supply to their members. It seems that some pharmacists have expressed dissatisfaction with the proposals on the ground that to supply an unknown quantity of drugs at a fixed rate would be an unbusinesslike transaction; but their opposition appears to have been based on the erroneous impression that they were to be paid so much per customer, whereas, of course, they will be paid for the goods they actually supply.

### THIS WEEK'S DRUG MARKET.

Few changes in the values of drugs have taken place during the week and business has been quiet. There is practically no alteration in the market position of opium, but the price tendency continues to be in an upward direction; morphine also tends dearer and makers of codeine have announced an advance in their quotations. Oil of almonds is slightly lower in price. Menthol continues to rise in value. The Cod-liver Oil market is dull and prices have a lower tendency. Eucalyptus oil is rather dearer and in good demand.

